# Effects of Candesartan on Electrical Remodeling in the Hearts of Inherited Dilated Cardiomyopathy Model Mice

**DOI:** 10.1371/journal.pone.0101838

**Published:** 2014-07-07

**Authors:** Fuminori Odagiri, Hana Inoue, Masami Sugihara, Takeshi Suzuki, Takashi Murayama, Takao Shioya, Masato Konishi, Yuji Nakazato, Hiroyuki Daida, Takashi Sakurai, Sachio Morimoto, Nagomi Kurebayashi

**Affiliations:** 1 Department of Cellular and Molecular Pharmacology, Juntendo University Graduate School of Medicine, Tokyo, Japan; 2 Department of Cardiovascular Medicine, Juntendo University Graduate School of Medicine, Tokyo, Japan; 3 Department of Physiology, Tokyo Medical University, Tokyo, Japan; 4 Department of Physiology, Faculty of Medicine, Saga University, Saga, Japan; 5 Department of Clinical Pharmacology, Graduate School of Medical Sciences, Kyushu University, Fukuoka, Japan; University of Buenos Aires, Faculty of Medicine. Cardiovascular Pathophysiology Institute, Argentina

## Abstract

Inherited dilated cardiomyopathy (DCM) is characterized by dilatation and dysfunction of the ventricles, and often results in sudden death or heart failure (HF). Although angiotensin receptor blockers (ARBs) have been used for the treatment of HF, little is known about the effects on postulated electrical remodeling that occurs in inherited DCM. The aim of this study was to examine the effects of candesartan, one of the ARBs, on cardiac function and electrical remodeling in the hearts of inherited DCM model mice (*TNNT2* ΔK210). DCM mice were treated with candesartan in drinking water for 2 months from 1 month of age. Control, non-treated DCM mice showed an enlargement of the heart with prolongation of QRS and QT intervals, and died at t_1/2_ of 70 days. Candesartan dramatically extended the lifespan of DCM mice, suppressed cardiac dilatation, and improved the functional parameters of the myocardium. It also greatly suppressed prolongation of QRS and QT intervals and action potential duration (APD) in the left ventricular myocardium and occurrence of ventricular arrhythmia. Expression analysis revealed that down-regulation of Kv4.2 (*I_to_* channel protein), KChIP2 (auxiliary subunit of Kv4.2), and Kv1.5 (*I_Kur_* channel protein) in DCM was partially reversed by candesartan administration. Interestingly, non-treated DCM heart had both normal-sized myocytes with moderately decreased *I_to_* and *I_Kur_* and enlarged cells with greatly reduced K^+^ currents (*I_to_*, *I_Kur_ I_K1_* and *I_ss_*). Treatment with candesartan completely abrogated the emergence of the enlarged cells but did not reverse the *I_to_*, and *I_Kur_* in normal-sized cells in DCM hearts. Our results indicate that candesartan treatment suppresses structural remodeling to prevent severe electrical remodeling in inherited DCM.

## Introduction

Inherited dilated cardiomyopathy (DCM) is a progressive disease characterized by dilatation and dysfunction of the ventricles, and often results in heart failure (HF) or sudden cardiac death (SCD) from lethal arrhythmia [1]–[2]. It has recently become clear that gene mutations in various cytoskeletal and sarcomeric proteins lead to weaknesses in the systems involved in force production, which can contribute to the development of DCM [3]. Mortality of DCM patients remains high, and the only treatment for DCM patients with severe HF symptoms is heart transplantation. In addition to HF development, electrical remodeling, which is accompanied by prolongation of the action potential duration (APD), can also be observed in DCM hearts and is thought to be related to increased arrhythmogenicity [4]–[5].

Recently, angiotensin II receptor blockers (ARBs) have been used for the treatment of HF. A growing body of evidence shows that ARBs inhibit cardiac hypertrophy, structural remodeling and ventricular arrhythmias in HF and thereby reduce cardiac morbidity and mortality [6]–[9]. However, their effects on inherited DCM are not well known. Because data in humans are confounded by various environmental and genetic factors, investigations with animal models of inherited DCM are required.

Among various gene mutations in inherited DCM, the deletion mutation ΔK210 in cardiac troponin T is a recurrent DCM-causing mutation that has been identified worldwide [10]–[11]. This mutation causes a lowered Ca^2+^ sensitivity in force generation of cardiac myofilaments [11]. A knock-in mouse model carrying this mutation was created by Morimoto and colleagues [12]–[14]. These mice closely recapitulate the phenotypes of human DCM, and previous study of this DCM model mouse indicated that down-regulation of various K^+^ channels is one of the causes of lethal arrhythmia and SCD [13].

The aim of this study was to examine whether candesartan, one of the ARBs, would have beneficial effects on cardiac function and electrical remodeling in inherited DCM using the ΔK210 knock-in mouse model. Our results indicate that early initiation of candesartan treatment dramatically expands lifespan, preserves cardiac function, suppresses cardiac enlargement as well as cellular enlargement, and attenuate electrical remodeling. Importantly, the suppressive effects of candesartan on electrical remodeling proved to be associated with the preventive effect on structural remodeling.

## Materials and Methods

### Animal model

All experiments were approved and carried out in accordance with the guidelines of the Committee for Animal Experimentation of Juntendo University (approval number 240001). Knock-in mice with the deletion mutation Lys-210 in their endogenous cardiac troponin T gene (*Tnnt2* ΔK210) were used as DCM model animals [12]. These mice had been backcrossed with the C57BL/6J line for at least 10 generations, and were maintained under specific pathogen-free conditions. Mixed-gender homozygous mutant and wild-type (WT) mice were obtained by crossing heterozygous mutant mice, and were used as DCM and non-DCM models, respectively.

### Drug administration

Candesartan cilexetil (TCV116), an ARB, was kindly provided by Takeda Chemical Industries, Ltd (Osaka, Japan). Candesartan was dissolved in drinking water for the mice. DCM mice were treated with candesartan (1 or 3 mg/kg/day) from 1 month of age. DCM mice at 3 months of age (after 2 months of treatment with candesartan, 3 mg/kg/day) and age-matched untreated DCM and WT mice were mainly used in this study. Hydralazine hydrochloride was obtained from Sigma-Aldorich Co. and administered in drinking water at 20 mg/kg/day.

### Electrocardiography (ECG), echocardiography, and blood pressure measurements

ECG recording of conscious mice was performed every 2 weeks from 1 month of age using an ECGenie (Mouse Specifics, Inc., Quincy, MA, USA), which non-invasively detected signals from the palmar and plantar aspects of the feet using footplate electrodes [13]. QT, RR, PR, and QRS intervals and heart rate (HR) were quantified. A HR-Corrected QT (QTc) interval was calculated with a modified Bazett correction formula for mice, QTc = QT/(RR/100)^1/2^ [15]. Telemetric ECG recordings were carried out using an implantable telemetry system with ECG transmitters (ETA-F10; Data Sciences International, St. Paul, MN, USA), which was implanted in mice at 2 months of age under isoflurane anesthesia [13]. ECG records were analyzed with ECG Analysis software (AD Instruments Japan, Nagoya, Japan).

The left ventricular end-systolic dimension (LVESD), end-diastolic dimension (LVEDD), fractional shortening (FS) and ejection fraction (EF) were measured by transthoracic echocardiography (Sonos 5500; Philips Electronics Co. Amsterdam, The Netherlands). Blood pressure was measured by a non-invasive tail-cuff system (Softron, Tokyo, Japan).

### Determination of wheel running activity

To estimate the extent of HF in DCM mice, physical activity was evaluated by measuring voluntary wheel running activity [13], [16]. Mice at 1.5 months or later were housed with free access to a running wheel (6 cm radius; Mini Mitter Co. Inc. OR, USA) for 2 days every 10 days, and their running activities (rounds/day) were measured.

### Determination of heart-to-body and lung-to-body weight ratios and plasma angiotensin II (Ang II) levels

Mice were deeply anesthetized with pentobarbital sodium (100 mg/kg i.p.), and their hearts and lungs were excised, rinsed in Krebs solution, and then weighed. Lung weight/body weight (LW/BW) and heart weight/body weight (HW/BW) ratios were calculated. To determine plasma Ang II levels, mice were euthanized and blood samples were obtained from the ventricles. The plasma concentration of Ang II was determined by a double antibody radioimmunoassay at Asuka Pharma Medical Co. Ltd (Tokyo, Japan).

### Solutions and reagents used in experiments with myocardium

Normal Krebs solution for myocardial experiments consisted of 120 mM NaCl, 5 mM KCl, 25 mM NaHCO_3_, 1 mM NaH_2_PO_4_, 2 mM CaCl_2_, 1 mM MgCl_2_ and 10 mM glucose, and was saturated with 95% O_2_ and 5% CO_2_. High-K^+^ Krebs solution used for muscle preparation consisted of 25 mM KCl instead of 5 mM. Di-4-ANEPPS was obtained from Invitrogen/Molecular Probes (Eugene, OR, USA).

### Optical determination of APD in isolated myocardium

The APD in myocardium was optically determined as described previously [13], [17]. Briefly, muscles of the left ventricle (LV) were loaded with di-4-ANEPPS and mounted in a chamber on the stage of an inverted microscope equipped with a Nipkow disc confocal system (CSU22; Yokogawa, Tokyo, Japan) and W-view/EM-CCD camera system (Model 8509; Hamamatsu Photonics, Hamamatsu, Japan). Myocardium was field stimulated at 0.5 Hz in normal Krebs solution. Di-4-ANEPPS was excited by 488 nm laser light and fluorescence images at 525 and 620 nm were simultaneously captured, and ratio images were then calculated. Membrane potential signals were obtained at 3.67 ms intervals. The APD_50_ (i.e., the time at which the down-stroke of the action potential (AP) had recovered 50% toward the baseline) was obtained as the activation minus the repolarization time points. Experiments were carried out at 25–27°C.

### Real-time RT-PCR

Total RNA was isolated from the LV using an RNeasy Fibrous Tissue Mini Kit (Qiagen). First strand cDNA was synthesized using a High-Capacity RNA-to-cDNA kit (Applied Biosystems) [13]. The expression of genes encoding Kv1.5, Kv2.1, Kv4.2, Kir2.1, Kir2.2, and KChIP2 was assessed by quantitative real-time PCR analysis using Fast SYBR Green Master Mix (Applied Biosystems). The primers used for real-time PCR have been described previously [13]. PCR was carried out using an 7500 Fast Real Time PCR System (Applied Biosystems). mRNA expression of each gene was normalized to that of the glyceraldehyde 3-phosphate dehydrogenase (GAPDH) gene.

### Western blot analysis

Membrane protein samples were analyzed by western blotting as described elsewhere [13]. The following antibodies were used: rabbit monoclonal IRK1 (Kir2.1) antibody (GTX62777, GeneTex), rabbit polyclonal anti-KCNB1 (Kv2.1) (SAB2101203, Sigma-Aldorich), rabbit polyclonal anti-Kv4.2 (APC-023; Alomone labs), rabbit polyclonal KChIP2 antibody (GTX116483, GeneTex), goat polyclonal anti-Kv1.5 (sc-11679; Santa Cruz Biotechnology), and mouse monoclonal anti-β-actin (ab8226; Abcam). The densities of specific bands were determined by MultiGauge software (Fujifilm, Tokyo, Japan) and normalized to that of the β-actin band.

### Whole-cell clamp recording

Single cells were isolated from the ventricles of mice using an established enzymatic method [18]. After isolation, the cells were whole-cell clamped in the voltage-clamp mode using a patch-clamp amplifier (Axopatch200B; Molecular Devices, Union city, CA, USA). Patch-pipettes (1.7–2.3 MΩ) were pulled from thin-wall glass capillaries (GC150TF-7.5; Harvard Apparatus, Holliston, MA, USA), and series resistance (2.5–5 MΩ) was compensated by 75–80% electronically. Data acquisition and analysis were performed using pClamp 10 software suit and a DigiData 1321A signal interface (Molecular Devices). All voltage data were corrected for a liquid junction potential of −10 mV assumed at the pipette tip. All measurements were carried out at room temperature (24–26°C). Ventricular cells were perfused with a bath solution consisting of 140 mM NaCl, 5.4 mM KCl, 1.8 mM CaCl_2_, 0.5 mM MgCl_2_, 0.33 mM NaH_2_PO_4_, 0.1 mM CdCl_2_, 11 mM glucose, and 5 mM HEPES-NaOH (pH 7.4). The pipette (intracellular) solution consisted of 110 mM K-aspartate, 30 mM KCl, 5 mM Mg-ATP, 0.1 mM Tris GTP, 4 mM BAPTA, and 20 mM HEPES-KOH (pH 7.2).

To record *I_K1_* and *I_ss_*, 2 s test pulses were applied to the cells every 4 s from a holding potential of −80 mV. To record *I_to_* and *I_Kur_*, 1 s test pulses with or without a prepulse (60 ms to −20 mV) were applied every 10 s from the holding potential. We confirmed that I_Kur_ was inhibited by 100 µM 4-aminopyridine (4AP) but not by the inactivating prepulse at 25°C, whereas I_to_ was inactivated by the prepulse but not by 4AP. Detailed procedures for isolation of each K^+^ current are described elsewhere [13], [19].

### Determination of cell length in isolated single myocytes

Isolated single cells on a laminin-coated glass bottom dish were incubated in a Ca^2+^ free Tyrode solution (140 mM NaCl, 5.4 mM KCl, 0.33 mM NaH_2_PO_4_, 0.5 mM MgCl_2_, 5.5 mM glucose and 5 mM HEPES, pH 7.4) and quickly fixed with a 4% paraformaldehyde (PFA) in phosphate buffered saline (PBS). Alternatively, concentrated single cells in the Ca^2+^ free Tyrode solution (∼50 µL) were fixed by adding ∼1 mL of 4% PFA in PBS. Both methods gave the same results. Fixed cells in a glass-bottom dish were observed with an inverted microscope and their images were captured. Cell lengths were measured with the AquaCosmos software (Hamamatsu Photonics, Hamamatsu, Japan). Cells showing contracture, which were distinguishable by collapsed shape or shortened sarcomere length, were omitted from the measurements.

### Statistics

Data are presented as the mean ± SEM unless indicated otherwise. Mean values were compared by one-way analysis of variance (ANOVA) followed by the post-hoc Kruskal-Wallis multiple comparison test for three groups. Two-way ANOVA followed by the Bonferroni post-test was used for comparison of multiple groups in a x-y relationship. P-values of <0.05 were considered significant.

## Results

### Effects of candesartan on survival rate, and cardiac histology and functions

**Figure 1:**
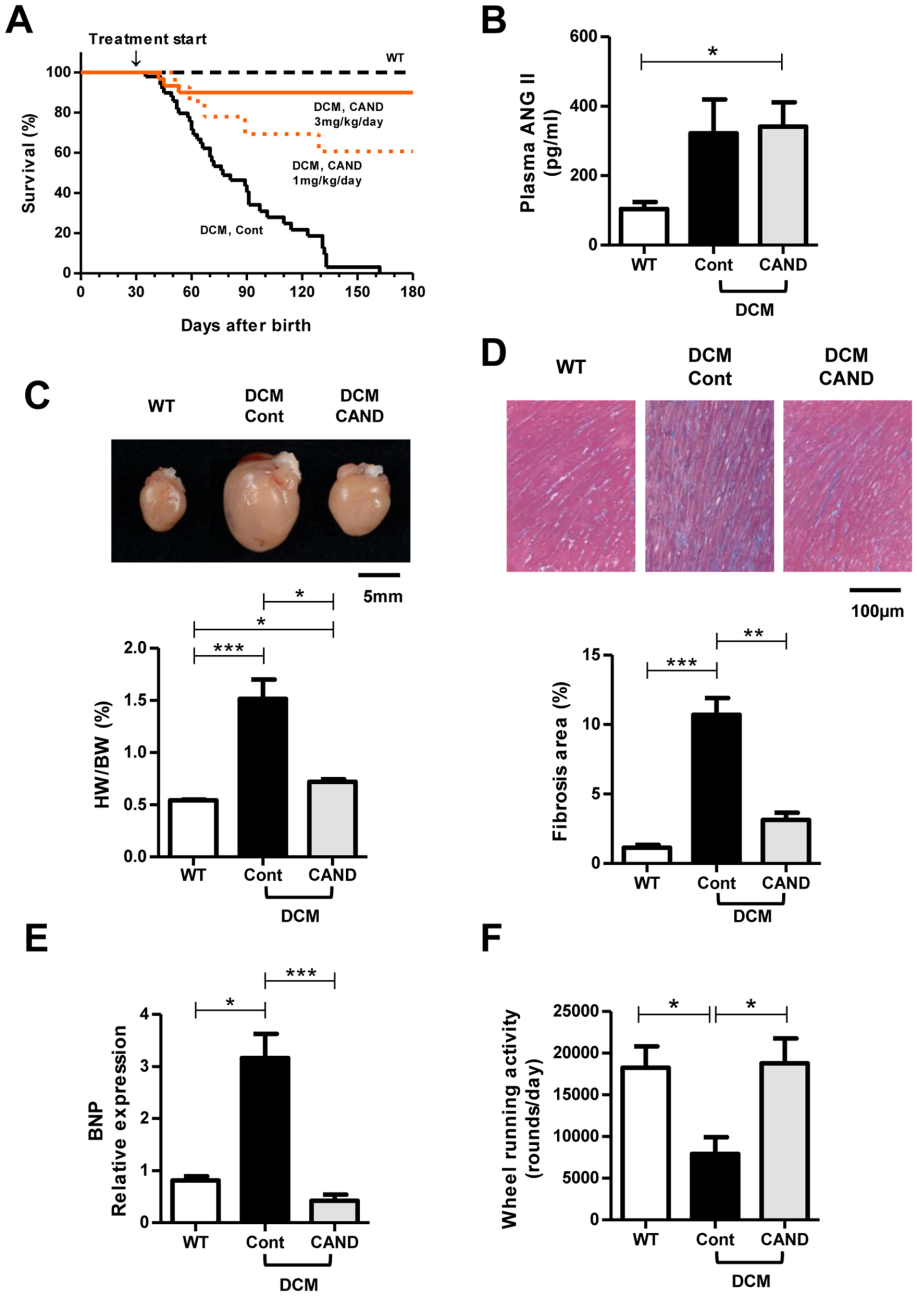
Effects of candesartan on survival rate, and cardiac histology and functions. ***A.*** Survival curves of control DCM (Cont) (n = 49), candesartan-treated DCM (CAND) (1 mg/kg/day: n = 14, 3 mg/kg/day: n = 30) and non-treated WT mice (n = 34). ***B.*** Plasma Ang II level (WT: n = 6, Cont: n = 5, CAND: n = 6). ***C.*** Typical gross morphology of hearts (upper image) and average HW/BW ratio (lower panel) (WT: n = 11, Cont: n = 11, CAND: n = 12). ***D.*** Histology of the LV myocardium. Connective tissues were stained blue with Azan. Upper images: typical images. Lower panel: quantitative analysis of the fibrotic area in the myocardium (three hearts in each group). ***E.*** Gene expression level of BNP in the LV (WT: n = 10, Cont: n = 15, CAND: n = 12). ***F.*** Wheel running activity (WT:n = 11, Cont: n = 10, CAND: n = 10). Data are the mean ± SEM. ^*^P<0.05, ^**^P<0.01, ^***^P<0.001.

In this study, treatment of DCM mice with candesartan was started at 1 month of age when their death rate was still low [13], [16]. Fig. 1A shows the Kaplan-Meier survival curve of DCM mice treated with candesartan (1 or 3 mg/kg/day) and the untreated control. In control DCM mice, t_1/2_ was approximately 70 days after birth (corresponding to 40 days of treatment) as described previously [12]–[13]. In contrast, the survival rates of candesartan-treated DCM mice were dramatically improved in a dose-dependent manner. Notably, in the group treated with 3 mg/kg/day, about 8% of mice died within 1 month after initiation of the treatment, but thereafter they rarely died until 6 months of age. In the following experiments, we analyzed DCM mice treated with 3 mg/kg/day.

First, we compared blood pressure to confirm the effect of candesartan in DCM mouse. The blood pressure in untreated DCM mice was already significantly lower than that in WT mice, and treatment with candesartan further reduced the blood pressure (Table 1). Plasma levels of Ang II were elevated in untreated DCM mice (Fig. 1B), indicating an increase of Ang II production. Plasma Ang II was also high in candesartan-treated DCM mice, which might be caused by production of plasma renin because of reduced blood pressure [20]–[21].

**Table 1:** In vivo parameters of WT and DCM mice with or without candesartan treatment.

	WT	DCM	
Treatment	-	-	Candesartan (3 mg/kg/day)
No. of mice	11	11	12
age, days	90.4±2.3	89.6±3.5	91.1±1.9
BW, g	23.6±1.0	22.4±0.7	23.5±1.1
HW, g	0.13±0.00	0.34±0.04***	0.17±0.01†††
LW, g	0.13±0.00	0.22±0.03**	0.13±0.01††
HW/BW, %	0.54±0.01	1.51±0.19***	0.72±0.02†††
LW/BW, %	0.56±0.02	1.00±0.14**	0.57±0.03†††
Blood pressure			
No. of mice	5	6	4
SBP, mmHg	113±3	99±5***	69±3* †††
DBP, mmHg	74±5	62±5	31±4*** ††
TTE			
No. of mice	10	11	12
HR, bpm	726.6±9.1	608.0±39.1**	712.5±12.5††
LVESD, mm	1.35±0.04	3.70±0.21***	1.85±0.09* †††
LVEDD, mm	2.82±0.05	4.58±0.22***	3.10±0.05†††
FS, %	52.2±1.6	19.6±1.3***	40.4±2.4*** †††
EF, %	84.5±1.3	40.2±2.5***	71.6±3.0* †††
ECG			
No. of mice	11	9	12
HR, bpm	730.5±7.8	591.7±46.6**	715.6±10.9††
PQ, msec	32.0±0.3	35.8±0.8**	32.4±0.8††
QRS, msec	10.2±0.2	18.6±0.9***	12.8±0.1*** †††
QT, msec	17.8±0.2	33.4±1.0***	19.5±0.5* †††
QTc, msec	19.3±0.2	27.6±2.2***	21.5±0.2* †††

DCM mice treated with candesartan (3 mg/kg/day) from 1 to 3 months of age and age-matched untreated control DCM and WT mice were used. Blood pressure, surface ECG and trans-thoracic echocardiography (TTE) were measured. Heart weight, lung weight and body weight were measured after sacrifice. BW, body weight; HW, heart weight; LW, lung weight; SBP, systolic blood pressure: DBP, diastolic blood pressure; HR, heart rate; bpm, beat per min; LVESD, LV end-systolic dimension; LVEDD, LV end-diastolic dimension, FS, fractional shortening; EF, ejection fraction.

^*^P<0.05,

^**^P<0.01,

^***^P<0.001 vs WT.

†† P<0.01,

††† P<0.001 vs control DCM.

Fig. 1C–F and Table 1 show the comparison of the properties of hearts from 3-month-old WT and DCM mice with or without candesartan treatment for 2 months. There were no significant differences in body weights between the three groups (Table 1). Fig. 1C shows the typical gross morphology of hearts (upper panel), and the average HW/BW ratios (lower panel). Untreated control DCM mice had considerably enlarged hearts compared with WT mice, and candesartan strongly suppressed the enlargement of the heart. However, it should be noted that the HW/BW in candesartan-treated mice was still higher (P<0.05) than that of WT mice.

Cardiac fibrosis was compared using Azan staining (Fig. 1D). The control DCM group showed advanced interstitial fibrosis, whereas candesartan greatly suppressed the fibrosis. The expression levels of brain natriuretic peptide (BNP) in the left ventricular myocardium, which have been associated with HF in humans and mice, were higher in control DCM mice than that in WT mice. The expression of BNP in the candesartan-treated group was completely reversed to the level in the WT group (Fig. 1E). Physical activity was measured using a voluntary running wheel to detect congestive HF (Fig. 1F) [13], [16]. The average wheel-running activity of control DCM mice was low at 3 months of age. In contrast, the candesartan group maintained a high running activity comparable to that of WT mice, suggesting that congestive HF did not developed in these mice. Importantly, candesartan treatment did not decrease daily motility despite the low blood pressure.

Echocardiographic analysis of untreated control DCM mice showed LV dilatation and systolic dysfunction as evidenced by an increased LVEDD and reduced LV EF, respectively (Table 1). Treatment with candesartan significantly decreased the LVEDD and increased the EF compared with those in control DCM mice (Table 1). Because previous report indicated that the decrease in the Ca^2+^ sensitivity of the myofilament in DCM heart was not improved by candesartan [22], above beneficial effects of candesartan are due to suppression of deterioration of heart. In summary, candesartan treatment greatly suppresses cardiac enlargement, fibrosis, and the development of HF in DCM mice without loss of physical activity.

### Effects of candesartan on ECG

**Figure 2:**
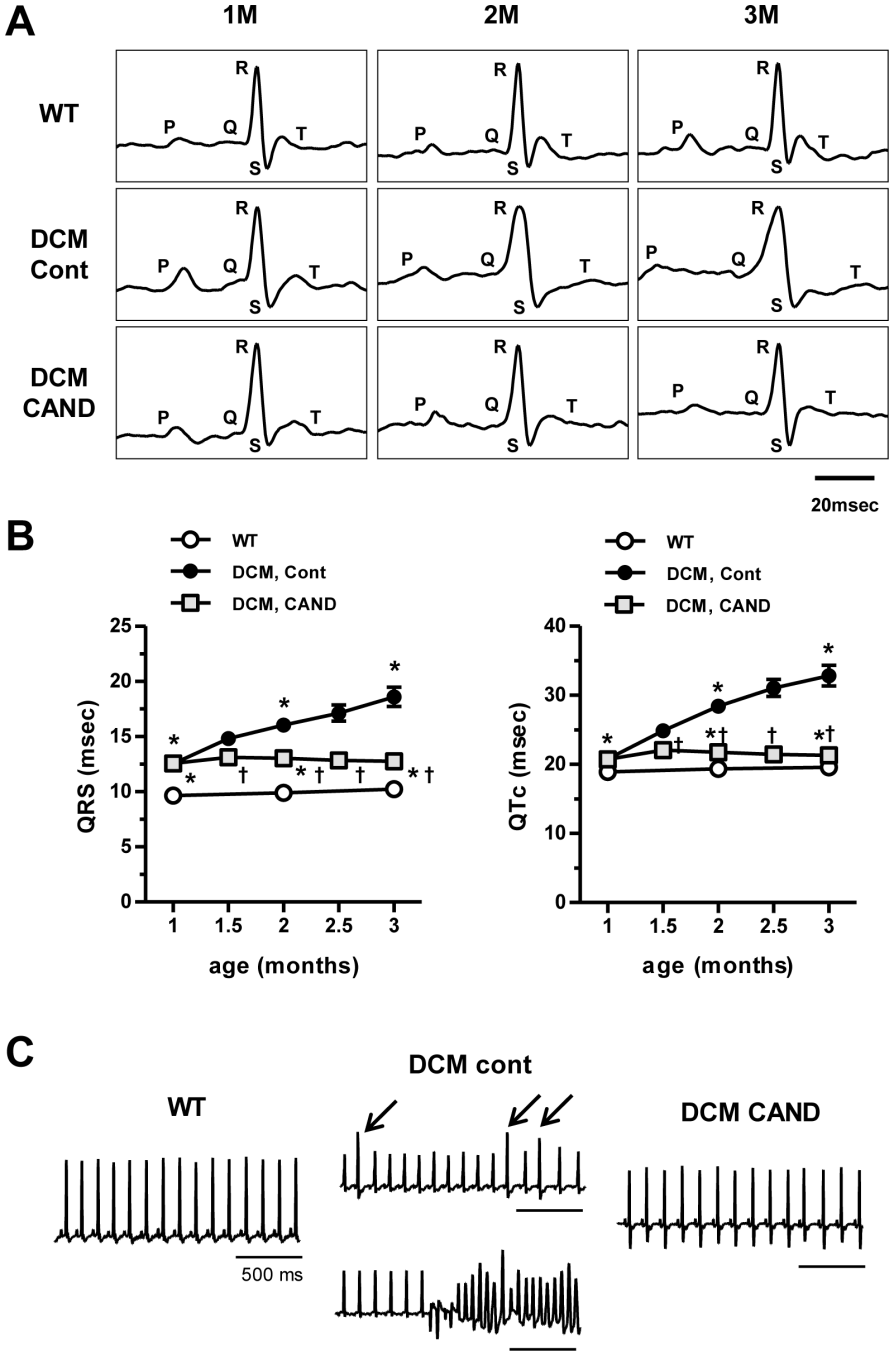
Effects of candesartan on ECG data. ***A*.** Typical traces of ECG in a time course. ***B.*** Comparison of QRS and QTc intervals in WT (n = 11), control (n = 11) and candesartan-treated DCM mice (n = 12). ^*^P<0.05 vs WT, ^†^P<0.05 vs control DCM. **C.** Typical ECG records obtained with a telemetric recording system. Left: normal ECG trace from WT mouse. Center: representative traces of PVCs (upper panel, arrows) and VT (lower panel) in control DCM mice. Right: representative normal ECG trace from candesartan-treated DCM mouse.

ECG records were non-invasively obtained from conscious mice every half month from 1 to 3 months of age. Heart rate (HR) and PQ, QRS, QT and QTc intervals in 3 month-old mice were summarized in Table 1. At 3 month-old, HR was significantly decreased in untreated DCM mice with increased variance; among 9 mice, 2 showed substantial decrease in HR to ∼350 beats per min (bpm) whereas others showed much less decrease (620–720 bpm). The HR was reversed to WT level by candesartan treatment (Table 1). Fig. 2B shows QRS and QTc intervals from 1 to 3 months of age. At 1 month of age before treatment with candesartan, QRS and QTc intervals were already significantly longer in DCM mice compared with those in WT mice (Fig. 2A and B). Thereafter, QRS and QTc intervals in control DCM mice were prolonged further. Treatment with candesartan completely suppressed further prolongation (Fig. 2A and B), but did not reverse to the WT level. The prolongation in QRS interval can be explained by an increase in conduction time in the ventricle probably because of cardiac enlargement and fibrosis, whereas the QTc prolongation suggests prolongation of APD.

There arises a question whether the suppressing effects of candesartan on prolongation of QRS and QTc intervals are due to direct inhibition of angiotensin receptor in heart or indirect action via further decreased blood pressure by vasodilation, which may reduce preload and afterload. To examine this, DCM mice were treated with a vasodilator, hydralazine, which lowers blood pressure independently of angiotensin receptor. Similarly to candesartan, hydralazine at 20 mg/kg/day further decreased blood pressure (from 99±5/62±5 mmHg (n = 6) to 74±2/47±3 mmHg (n = 6)) and increased HR (from 592±47 bpm to 722±7 bpm) in DCM mice. However, hydralazine treatment did not suppress the prolongation of QRS and QTc intervals in DCM mice (Figure S1 in File S1). Thus, the suppressive effects of candesartan on prolongation of QRS and QTc could not be attributed to the decreased blood pressure.

We also obtained ECG records using an implantable telemetry system with ECG transmitters with mice at 2–5 months of age until the battery of the transmitter stopped functioning (Fig. 2C). All untreated control DCM mice (n = 6) showed occasional ventricular arrhythmia including premature ventricular contraction (PVC), ventricular tachycardia (VT) (Fig. 2C, middle), and/or T wave alternans, and finally died with VT or Torsades des pointes within a ∼2.5 months of monitoring period as reported previously [12]–[13], [16]. In contrast, WT (n = 5) and candesartan-treated DCM mice (n = 6) showed no such ventricular arrhythmia and survived far beyond the monitoring period. These results suggest that some changes in electrical properties related to APD and arrhythmia may be suppressed by candesartan treatment. Because the differences in QRS and QTc intervals between DCM control and candesartan-treated mice were most clear after 2 months of treatment (at 3 months of age), comparisons were made at this age in the following experiments.

### Effects of candesartan on APD in isolated myocardium

**Figure 3:**
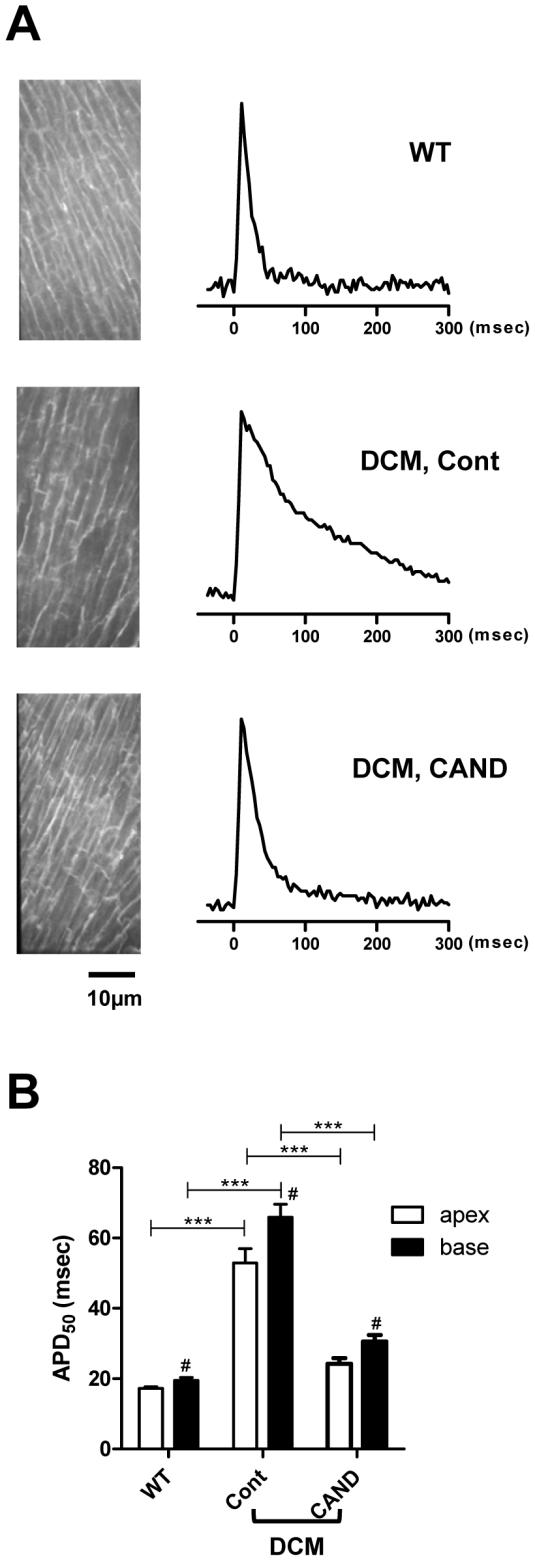
AP signals obtained from WT and DCM LVs. ***A.*** Representative AP signals recorded from the basal region of LVs of WT (top), control DCM (middle) and candesartan-treated DCM (bottom) mice stimulated at 0.5 Hz. ***B.*** Comparison of APD_50_ values in the basal and apical regions of LVs from WT (base: n = 10, apex: n = 12 from five hearts) control DCM (base: n = 31, apex: n = 35 from 6 hearts) and candesartan-treated DCM (base: n = 25, apex: n = 21 from four hearts) mice. ^***^P<0.001, ^#^P<0.05 vs the apex in the same group.

Because QT interval is closely related to APD, AP signals were optically obtained at apical and basal regions of the LV as described previously [13]. APD_50_ (APD at 50% repolarization) values were used because they are relatively stable compared with longer APD measurements such as APD_90_, which are more variable as they are affected by Na^+^-Ca^2+^ exchange activity. In the basal region of the WT LV, the average APD_50_ was approximately 20 ms, whereas that in the DCM LV was about 3.2-fold longer (64±4 ms) (Fig. 3A and B). In the candesartan-treated DCM LV, APD_50_ was significantly shorter (31±2 ms) than that in the control DCM. These results were consistent with QTc intervals in the three groups (Fig. 2 and Table 1). Comparison of APD_50_ at the apical region among the three groups gave similar conclusions. The spatial difference between basal and apical regions was largest in control DCM mice, and was much smaller in candesartan-treated DCM mice (Fig. 3B).

### Effects of candesartan on the gene and protein expression of K^+^ channels

The shortened APD in the myocardium of candesartan-treated DCM mice can be explained by increased repolarizing currents. APD has been reported to be influenced by *I_to_*, *I_Kur_*, *I_K1_* and *I_ss_* [19], and we have previously found that the expression of *I_to_*- and *I_Kur_*-related molecules is considerably decreased in DCM mice at 2 months [13]. In this study, we analyzed the expression levels of genes encoding the various ion channels that carry *I_K1_* (Kir2.1 and Kir2.2), *I_ss_* (Kv2.1), *I_to_* (Kv4.2 and the accessory subunit KChIP2), and *I_Kur_* (Kv1.5) in WT and DCM mice at 3 months.

**Figure 4:**
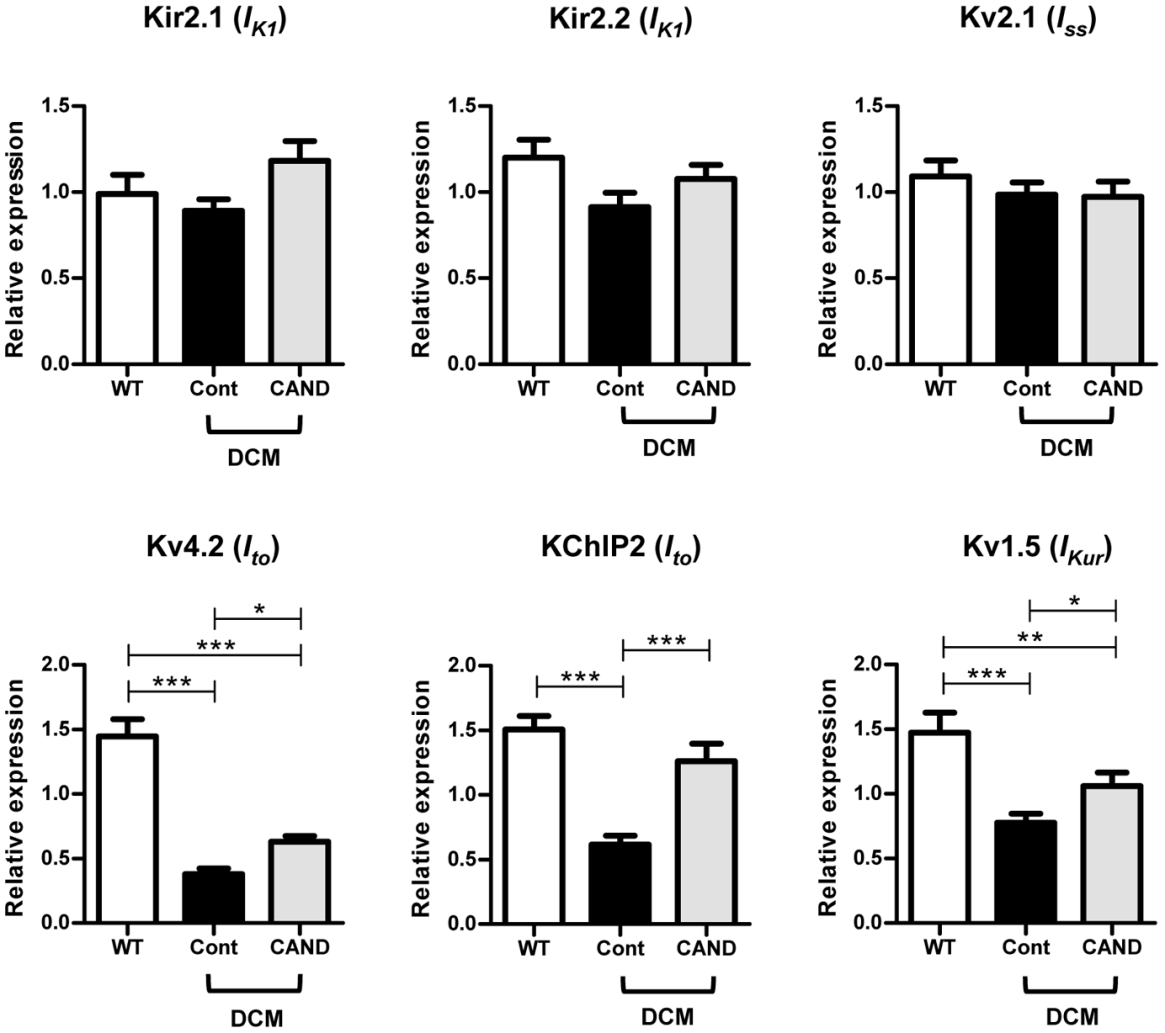
Expression levels of mRNA encoding the K^+^ channels and auxiliary subunits that contribute to the repolarizing phase of APs. Quantitative real-time PCR analysis was carried out with LVs from WT (n = 10), control (n = 15) and candesartan-treated DCM (n = 11) mice. The GAPDH gene was used as an internal control. ^*^P<0.05, ^**^P<0.01, ^***^P<0.001.

There was no significant difference in the expression levels of Kir2.1, Kir2.2 and Kv2.1 among the three groups. In contrast, the mRNA levels of Kv4.2, KChIP2, and Kv1.5 were significantly diminished in the untreated control DCM LV to about half or less of the WT level (Fig. 4). These results were consistent with our previous report [13]. Candesartan significantly, but only partially restored the expression of Kv4.2 and Kv1.5, and greatly restored the expression of KChIP2. To know whether the candesartan directly up-regulate these channels, WT mice were also treated with candesartan and gene expression levels were determined. As shown in Figure S2 in File S1, candesartan has no significant effects on any of Kir2.1, Kir2.2, Kv2.1, Kv4.2, KChIP2 and Kv1.5. These results suggest that candesartan does not directly up-regulate these channels but inhibit action of Ang II elevated in DCM mice.

**Figure 5:**
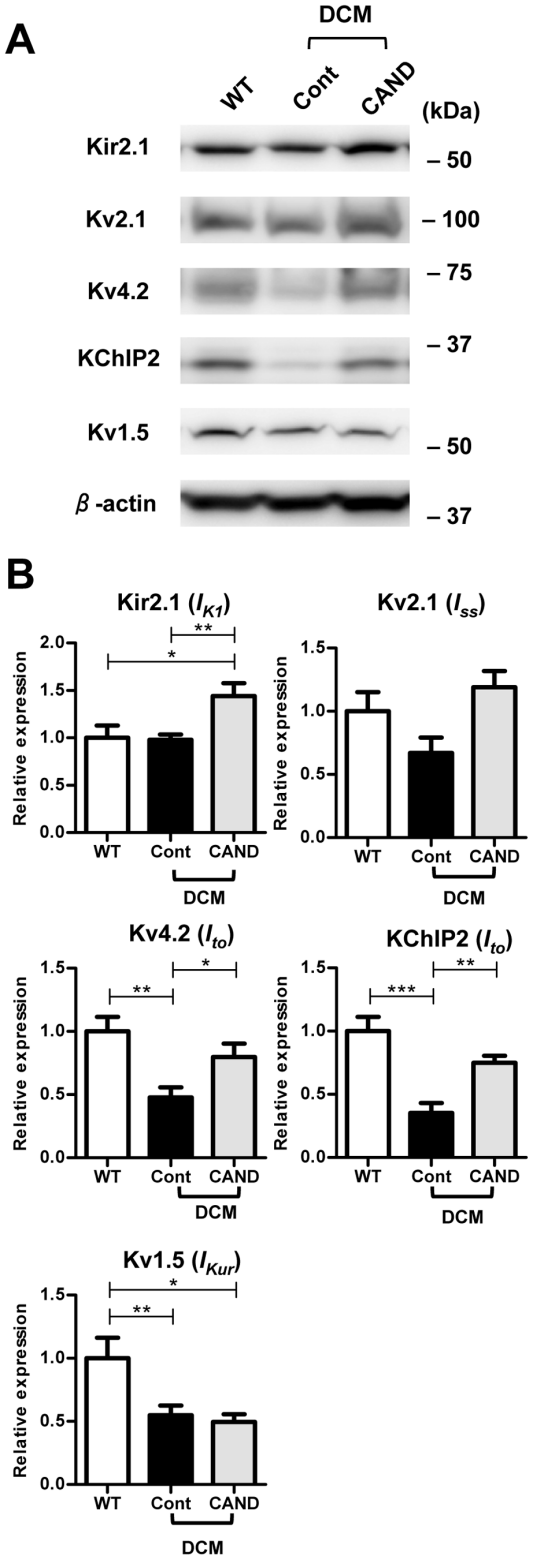
Western blot analysis of the K^+^ channels and auxiliary subunits in the LV. Membrane protein samples (50 µg) of WT (n = 6), control (n = 10) and candesartan-treated DCM (n = 7) LVs were separated by SDS-PAGE and western blot analysis was carried out. See Methods for details. ***A.*** Representative blots of individual experiments. ***B.*** Averaged expression levels. The relative expression levels for each protein were normalized to the average value for WT LVs. ^*^P<0.05, ^**^P<0.01, ^***^P<0.001.

Expression of channel proteins was verified by western blot analysis. Fig. 5 shows typical data (A) and the comparison of the averaged values of each protein level (B). There was no significant difference in the protein levels of Kir2.1 between WT and control DCM LVs, and candesartan moderately up-regulated Kir2.1 protein expression. The expression level of Kv2.1appeared to be reduced in control DCM and restored in candesartan treatment, but without significant difference. In contrast, Kv4.2 and KChIP2 levels were decreased in DCM LVs to ∼50% of those in WT LVs, and were considerably restored by candesartan treatment. These results are roughly correlated with the gene expression data. The level of Kv1.5 was also significantly decreased to about half of that in WT LVs, and candesartan had no significant effect on the expression. The reason for the discrepancy between gene and protein expression of Kv1.5 is unclear.

### Effects of candesartan treatment on K^+^ currents

**Figure 6:**
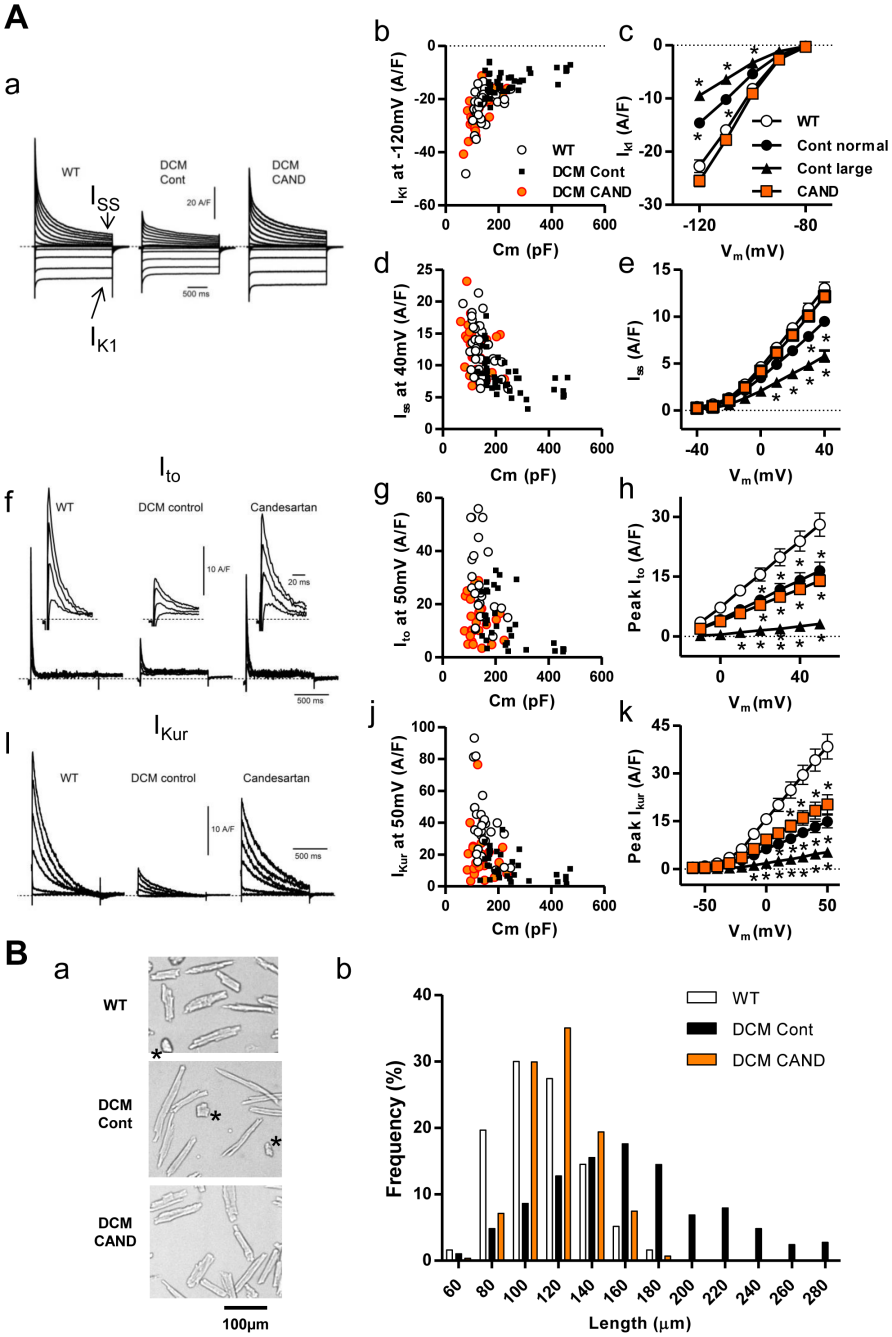
Whole-cell currents of major K^+^ channels which contribute to repolarization and the size distribution of ventricular myocytes. ***A.*** *I_K1_*, *I_ss_ I_to_*, *I_Kur_* of WT, control and candesartan-treated DCM cells. ***a.*** Representative current traces of whole cell currents activated by voltage steps (2 s, −120 to +40 mV in 10 mV increments) from a holding potential of −80 mV. *I_K1_* and *I_ss_* were measured as the steady-state amplitude of the whole-cell current at pulse end. ***b*** and ***d***, *I_K1_* and *I_ss_* in individual cells at the largest voltage step (−120 mV and +40 mV, respectively) were plotted against their Cm, which reflects the cell surface area. ***c*** and ***e***. Current-voltage (IV) relations of *I_K1_* and *I_SS_*, respectively. ***f*** *.* Representative current traces of *I_to_* activated at −10, +10, +30, and +50 mV (insets expand the peaks on a fast time-base). ***g.*** *I_to_* in individual cells at the largest voltage step, 50 mV. ***h.*** IV relations of *I_to_*. ***i.*** Representative current traces of *I_Kur_* activated at −50, −30, −10, +10, +30, and +50 mV. ***j.*** *I_Kur_* in individual cells at the voltage step of 50 mV. ***k.*** IV relations of *I_Kur_*. ***b***, ***d***, ***g*** and ***j***: WT (open circle, n = 25–34 from six hearts), Cont (filled square, n = 33–43 from eight hearts), CAND (orange circle, n = 26–36 from five hearts). ***c***, ***e***, ***h*** and ***k***: WT (open circle, n = 25–34), normal-Cm control DCM (Cm<250 pF, filled triangle, n = 14–23), large-Cm control DCM, (300 pF<Cm<500 pF, filled circle, n = 6–8), candesartan-treated DCM (orange square, n = 26–36), Dotted lines: zero-current level. ^*^P<0.001 vs WT. ***B*** *.* Length distributions of ventricular myocytes. ***a.*** Typical pictures of myocytes obtained with 10× objective lens. Images at higher magnifications were shown in Figure S4 in File S1. *Cells showing contracture, which were discernable from shrunken shape and shortened sarcomere length, were excluded from analysis. **b.** Histogram of longitudinal length of ventricular myocytes for WT (n = 310 from five heart), Cont (n = 290 from six heart) and CAND (n = 294 from five hearts).

We next examined the effects of candesartan treatment on major K^+^ currents (*I_K1_*, *I_ss_*, *I_to_*, and *I_Kur_*) in ventricular myocytes [13], [19]. As we noticed that the membrane capacitance (Cm) of untreated control DCM myocytes was often large, we carried out current measurements from randomly selected cells that allowed successful patch clamp recording except for cells with a Cm of >500 pF, which are thought to be difficult to clamp voltage. Fig. 6A-a shows typical whole cell K^+^ currents in myocytes activated by 2 s square pulses (−120 to +40 mV) from a holding potential of −80 mV. The amplitude of outward whole-cell current activated by depolarizing pulse was reduced in control DCM cells compared with WT cells, and was considerably restored in cells from candesartan-treated DCM mice, consistent with the results of APD_50_ measurements.

Because there was a large Cm variation in control DCM cells, currents in individual cells at the largest voltage step (−120 mV for *I_K1_*, +40 mV for *I_SS_*, +50 mV for *I_to_* and *I_Kur_*) were plotted against their Cm, which reflects the cell surface area (Fig. 6-b, d, g, j). Since the largest Cm for WT myocytes was approximately 250 pF, I–V relationships of control DCM cells were plotted for two groups, cells with normal Cm (Cm<250 pF) and cells with large Cm (300 pF<Cm<500 pF) (Fig. 6-c, e, h, k). Interestingly, no large-Cm cells were detected in population of candesartan-treated myocytes.

The inward rectifier K^+^ current (*I_K1_*) was measured as a steady-state current activated by hyperpolarizing pulses (Fig. 6A-a). When comparisons were made between normal-Cm cells, *I_K1_* was smaller in control DCM cells (67%) compared with that in WT cells (Fig. 6A-c). In large-Cm DCM cells, *I_K1_* was further reduced (43%). In contrast, candesartan treatment completely reversed *I_K1_* to the level in WT cells (Fig. 6A-c). The *I_ss_* was isolated as the steady-state whole-cell current at depolarizing pulse end (Fig. 6A-a). The amplitude of the *I_ss_* in normal-Cm control DCM cells was slightly smaller (73%) than that in WT cells, and that in large-Cm cells was further reduced (45%). The amplitude of the *I_ss_* in candesartan-treated cells was much closer to the WT level (Fig. 6A-e).

Both *I_to_* and *I_Kur_* are transient outward currents, but they exhibit different sensitivities to 4 aminopyridine (4AP) and an inactivating prepulse [13], [19]. *I_to_* was measured as the prepulse-sensitive outward current in the presence of 4AP, while *I_Kur_* was measured as the 4AP-sensitive outward current activated by depolarizing test pulses with the prepulse as described previously [13]. *I_to_* in control DCM cells with a normal Cm had significantly smaller peak amplitudes (∼50%) than those in WT cells (Fig. 6A-f–h). To our surprise, control DCM cells with a large Cm appeared to have much lower current densities (∼11%) (Fig. 6A-g, h). The *I_to_* in candesartan-treated cells was approximately 50% of WT level, similar to that in normal-Cm control DCM cells (Fig. 6A-k). *I_Kur_* in normal-Cm control DCM cells was also significantly smaller (37%) than that in WT cells (Fig. 6A-j, k). *I_Kur_* in large-Cm DCM cells was further reduced (∼13%) compared to normal-Cm cells (Fig. 6A-j, k). *I_Kur_* in candesartan-treated DCM cells was similar to that in normal-Cm control DCM cells (50% of WT).

Because measurements of current densities for *I_to_* and *I_Kur_* in large-Cm cells might be less accurate by distortion of the time courses of transient outward currents, we examined current kinetics (time to peak from onset of voltage step and decay time from 100 to 50% of peak) and plotted against Cm (Figure S3 in File S1). Since the time to peak and decay time were similar over Cm range of 80–500 pF, values for these K^+^ currents in large-Cm cells were considered acceptable. In summary, the above current measurements indicate that current densities of all four components decrease in both normal- and large-Cm control DCM cells. Candesartan completely restores *I_K1_* and *I_SS_* with no significant effect on *I_to_* or *I_Kur_* in DCM cells with normal Cm. More importantly, however, candesartan suppresses emergence of large-Cm cells, which have very low densities of *I_to_* and *I_Kur_*.

### Effects of candesartan treatment on cell-length distribution of ventricular myocytes in DCM heart

The presence of large-Cm cells with low densities of K^+^ currents may account for the data on mRNA/protein expression, APD and current measurements. However, the fraction of the large-Cm cells appeared to be smaller for successful patch-clamp recordings than that of the *in vivo* population in the DCM myocardium because making the whole-cell configuration with them was difficult by unstable sealing. To determine the actual size distribution of isolated ventricular myocytes, myocytes were fixed with PFA and cell lengths were determined (see Materials and methods, Fig. 6Ba, Figure S4 in File S1). The frequency distributions of longitudinal cell lengths were plotted in Fig. 6B-b. WT myocytes ranged between 52 and 184 µm (mean±SD: 111±25 µm, n = 310), whereas untreated control DCM cells ranged between 59 and 290 µm with a larger standard deviation (161±50 µm, n = 290, P<0.001 vs WT). Thus, the distribution of cell size increased in the control DCM heart, and about 27% of myocytes were out-of-range compared with the WT cell distribution. In contrast, candesartan-treated DCM cells ranged from 64 to 182 µm (119±21 µm, n = 294, P<0.05 vs WT, P<0.001 vs control DCM). Although the mean size of candesartan-treated DCM cells was significantly larger than that of WT cells, we did not detect any enlarged cells (>190 µm). It should be noted that hydralazine treatment did not suppress the emergence of enlarged cells (data not shown). These results confirm that candesartan treatment prevent the emergence of enlarged cells in DCM hearts.

## Discussion

The beneficial effects of ARBs on chronic HF have been well established in animal models [6]–[8], but the effects on electrical remodeling in inherited DCM have not been well known because of the lack of appropriate models. Thus far, only a few studies have revealed the effects of ARBs on DCM using animal models [23], and these studies have not assessed the effects of ARBs on electrical remodeling in inherited DCM. In the present study, we found that candesartan improved the survival rate and cardiac systolic function, and suppressed progression of cardiac dilation and fibrosis using a DCM mouse model with the ΔK210 mutation in *TNNT2*, which closely recapitulates the human phenotype [12]–[14], [16]. In addition to the above effects, candesartan suppressed occurrence of ventricular arrhythmia as well as various changes in the electrical properties in DCM hearts. These effects are probably due to the direct inhibition of angiotensin receptor in heart, but not due to indirect action via decreased blood pressure, because vasodilator hydralazine did not show suppressive effects on cardiac enlargement or prolongation of QT/APD. This is consistent with a previous report that many of β-adrenoreceptor blocker, which reduce mean blood pressure, had no suppressive effects on cardiac enlargement or prolongation of QT [14]. Interestingly, the changes in electrical properties of DCM heart seem to be closely related to the emergence of enlarged cells, and candesartan dramatically prevented the emergence.

### Progression of multiple types of electrical remodeling in ΔK210 DCM hearts

Our previous study [13] and this study demonstrated that multiple types of progressive channel remodeling occur at different time points in the hearts of DCM model mice. (1) Some genes are already modulated in DCM heart from neonate when they show no symptoms of HF and very low mortality. For example, the mRNA level of Kv4.2 is significantly reduced in DCM heart in the neonatal period [13]. (2) Then the DCM mice start to display high mortality at around 2 months of age in parallel with prolongation of the QT interval and APD_50_ as well as a reduction in *I_Ku_*_r_/Kv1.5 and I_to_/Kv4.2 and KChIP2. However, most of DCM mice do not develop congestive HF at this age [13], [16]. (3) Thereafter, at around 3 months, their hearts enlarge further as they enter the congestive HF stage [13], [16]. Expression levels of Kv4.2, Kv1.5, and KChIP2 decrease further together with prolongation of the APD_50_. In addition, this study revealed that, at 3 months, *I_K1_* and *I_SS_* decreased without significant changes in mRNA or protein levels, which is probably because of post-translational changes that occurred in later stage of HF [23]–[25].

A prominent change in DCM heart was the emergence of enlarged cells with very low densities of K^+^ currents, which is probably related to cardiac dilation. The longitudinal length distribution of isolated myocytes at 3 months of age indicates that about 27% of myocytes were out-of-range compared with the WT cell distribution. If only longitudinal length increases in DCM heart, the enlarged cells would occupy 40% of total volume because cell volume is proportional to the length in this case. However, volume occupancy of the enlarged cells should be greater than 40% because heart weights of DCM mice were 2∼3 times greater than that of WT mice. Cell width and thickness of DCM cells may also be increased. Actually, enlarged cells with increased length and width were often observed at endocardium side in DCM myocardium (data not shown), although quantitative analysis was difficult at present. Another explanation is that enlarged cells might be lost during preparation of single cells because of their labile property. In addition, non-cardiac cells such as fibroblast might contribute to heart weight. In any event, the enlarged cells with low K^+^ currents may greatly contribute to prolonged APD, unstable repolarization and arrhythmia. Although there are differences in K^+^ current components between human and mice, similar changes in structural and electrical properties are likely to occur in human inherited DCM hearts.

### Effect of candesartan on various types of electrical remodeling

This study showed that many but not all types of electrical remodeling in DCM mice were ameliorated by candesartan treatment. *In vivo* and *in situ* measurements indicated that candesartan substantially suppressed prolongations of the QT interval and APD_50_. Interestingly, prolongation of these parameters occurred long before onset of congestive HF [13], [16] and the suppressive effect of candesartan was already obvious at early age (Fig. 2). Thus, candesartan inhibits effects of Ang II that elevates in DCM mice when their hearts are still compensated.

The most likely explanation for the shortened APD_50_ by candesartan treatment is restoration of the repolarizing K^+^ currents (*I_to_*, *I_Kur_*, *I_K1_*, and *I_ss_*) [19]. In particular, *I_to_* and *I_Kur_* underlie the early phase of myocardial AP repolarization, contributing to the APD_50_ and coordinated propagation of activity in the heart. In the hearts of candesartan-treated mice, enlarged cells were virtually absent. Instead, *I_to_* and *I_Kur_* were moderately lowered (∼50% of WT) in normal sized cells in candesartan-treated DCM heart. As a results, *in vivo* average of *I_to_* and *I_Kur_* in the candesartan-treated DCM heart can be higher than that in the control DCM heart which have enlarged cells with very low *I_to_* and *I_Ku_*_r_ (∼10∼20% of WT). In summary, suppression of the emergence of the enlarged cells with low K^+^ current density is one plausible explanation for the shortened QT interval and APD_50_ in the candesartan-treated DCM heart.

It is interesting that candesartan could not reverse the moderate decrements in *I_to_* and *I_Ku_*_r_ in normal sized DCM cells (∼50% of WT). DCM mice may be safe with such moderate decrease alone. Furthermore, this indicates that other molecules than Ang II also contribute to the down regulation of these channels. A wide range of signaling molecules including, Ang II, catecholamine and glucocorticoids, has been implicated in regulating long-term changes in expression of K^+^ channels [26]–[29]. Further studies are needed to understand the regulatory mechanisms of ion channel expression during progression of DCM disease.

In contrast to partial recovery of *I_to_* and *I_Kur_*, candesartan completely restored the decrements in *I_K1_* and *I_SS_*, which was not associated with changes in protein or mRNA levels. Because there were no differences in *I_K1_* and *I_SS_* between WT and DCM at 2 months [13], candesartan probably suppresses the post-translational reduction that occurs in the later stage of DCM. Thus, treatment of DCM mice with candesartan starting at 1 month completely prevented the reduction of *I_K1_* and *I_ss_*. These effects may also partially contribute to the suppression of the prolongation of QT intervals and APD_50_.

In addition to the reduction of K^+^ currents, another prominent change in DCM hearts was the pathologic proliferation of fibroblasts. The progression of fibrosis is associated with conduction disorder, arrhythmogenesis and the prolonged APD_50_ in the control DCM heart because fibroblast could electrically couple to cardiomyocytes [30]. Collectively, abrogation of such fibrosis by candesartan treatment also contributes to prevention of conduction disorder, APD prolongation and arrhythmogenicity in DCM mice.

### Candesartan-insensitive remodeling

We noticed both candesartan-sensitive and -insensitive remodeling in this model mouse. The candesartan-sensitive remodeling may be attributed to the renin-angiotensin system (RAS) because there was an elevation of the plasma Ang II concentration. A transgenic mouse model over-expressing the Ang II receptor, which shows a reduction of *I_to_*, *I_Kur_*, *I_K1_* and *I_ss_* [31], also supports this idea. On the other hand, the candesartan-insensitive electrical remodeling was probably because of mechanisms other than the RAS [24], [26]. For example, changes such as basal cardiac enlargement (Fig. 1) and prolongation of QRS and QT intervals (Fig. 2) occurring in the early stage [13], i.e., moderate decrements in Kv4.2 and Kv1.5 in early stage (Figs. 4–6), and an increment in Cav3.1 (data not shown), appear to be unaffected by candesartan. However, an alternative possibility is that the remodeling once developed in the early stage cannot be reversed by ARB treatment. Further studies are required to understand the detailed remodeling processes in the inherited DCM heart.

### Conclusion

In this study, we found that inhibition of changes in the later stage of DCM, i.e., cardiac enlargement, fibrosis and various changes in channel expression, contributes to the beneficial effect of ARBs. Our results suggest that the effect of candesartan on electrical remodeling is closely related to the suppressive effect on structural remodeling and that the early initiation of candesartan treatment is highly effective for prevention of disease in inherited DCM. Because candesartan treatment does not suppress voluntary exercise activity and hence preserves the quality of life, ARBs are an excellent candidate drug for treating inherited DCM patients.

## Supporting Information

File S1 **Figure S1. Effects of hydralazine on ECG data.** ***A*** **.** Typical traces of ECG from WT and Hydralazine treated DCM mice. ***B.*** Comparison of QRS (upper panel) and QTc (lower panel) intervals. Data with WT, untreated control and candesartan-treated DCM mice are same as those in Fig. 2B. n = 7 for hydralazine. ^*^P<0.05 vs WT, ^†^P<0.05 vs control DCM. **Figure S2. Effects of candesartan treatment on expression levels of mRNA encoding K^+^ channels and accessory subunit in WT left ventricle.** Quantitative real-time PCR analysis was carried out with LVs from control WT (n = 10) and candesartan-treated WT (n = 8) mice. The GAPDH gene was used as an internal control. **Figure S3. Current kinetics of** ***I_to_*** **(** ***A*** **and** ***C*** **) and** ***I_Kur_*** **(** ***B*** **and** ***D*** **).** Time to peak (*A* and *B*) and decay time (100 to 50% of peak) (*C* and *D*) are plotted against membrane capacitance. Cm was poorly correlated with current kinetics of *I_to_* or *I_Kur_*. **Figure S4. Typical images of myocytes obtained with 60× objective lens.** ***A.*** WT, ***B.*** Candesartan treated DCM. ***C.*** Control DCM.
(PDF)
