# Supplementary material for: Effects of Candesartan on Electrical Remodeling in the Hearts of Inherited Dilated Cardiomyopathy Model Mice
Source: PLoS One. 2014 Jul 7;9(7):e101838. doi: 10.1371/journal.pone.0101838 (PMC4084897; doi:10.1371/journal.pone.0101838)
Supplement: File S1 — Figure S1. Effects of hydralazine on ECG data. A . Typical traces of ECG from WT and Hydralazine treated DCM mice. B. Comparison of QRS (upper panel) and QTc (lower panel) intervals. Data with WT, untreated control and candesartan-treated DCM mice are same as those in Fig. 2B. n = 7 for hydralazine. *P<0.05 vs WT, †P<0.05 vs control DCM. Figure S2. Effects of candesartan treatment on expression levels of mRNA encoding K+ channels and accessory subunit in WT left ventricle. Quantitative real-time PCR analysis was carried out with LVs from control WT (n = 10) and candesartan-treated WT (n = 8) mice. The GAPDH gene was used as an internal control. Figure S3. Current kinetics of Ito ( A and C ) and IKur ( B and D ). Time to peak (A and B) and decay time (100 to 50% of peak) (C and D) are plotted against membrane capacitance. Cm was poorly correlated with current kinetics of Ito or IKur. Figure S4. Typical images of myocytes obtained with 60× objective lens. A. WT, B. Candesartan treated DCM. C. Control DCM. (PDF) [file pone.0101838.s001.pdf]

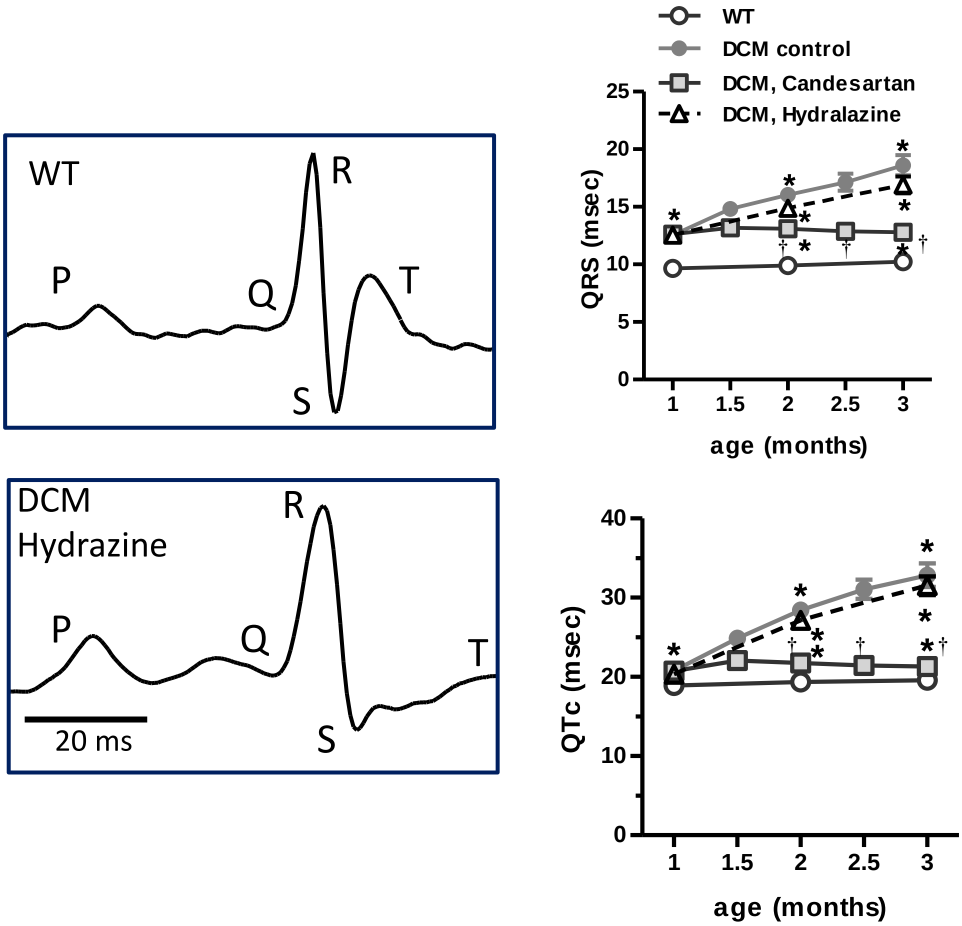

Figure S1 Effects of hydralazine on ECG data. ***A.*** Typical traces of ECG from WT and Hydralazine treated DCM mice. ***B.*** Comparison of QRS (upper panel) and QTc (lower panel) intervals. Data with WT, untreated control and candesartan-treated DCM mice are same as those in Fig. 2B. n=7 for hydralazine. $^{*}P < 0.05$ vs WT, $^{\dagger}P < 0.05$ vs control DCM.

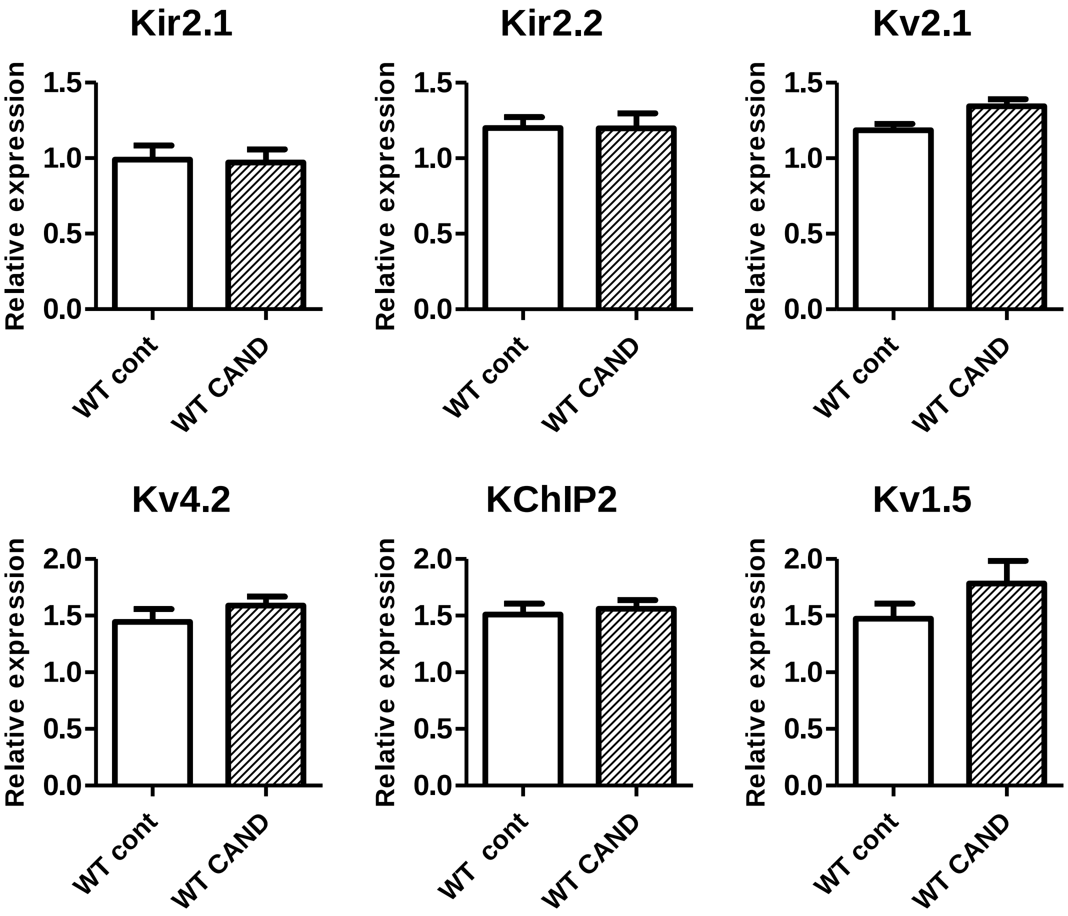

Figure S2 Effects of candesartan treatment on expression levels of mRNA encoding K$^+$ channels and accessory subunit in WT left ventricle. Quantitative real-time PCR analysis was carried out with LVs from control WT (n=10) and candesartan-treated WT (n=8) mice. The GAPDH gene was used as an internal control.

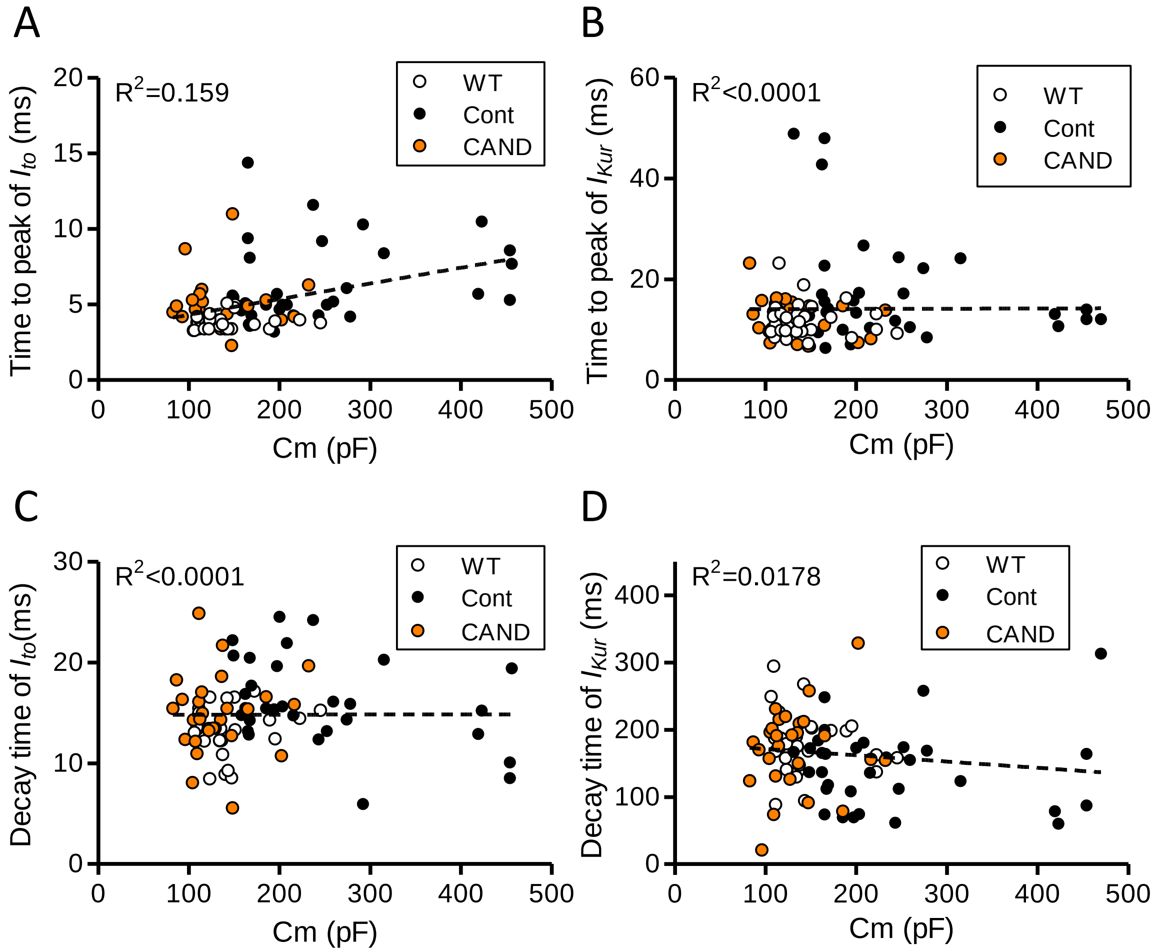

Figure S3. Current kinetics of I_{to} (A and C) and I_{Kur}(B and D). Time to peak (A and B) and decay time (100 to 50% of peak) (C and D) are plotted against membrane capacitance. Cm was poorly correlated with current kinetics of I_{to} or I_{Kur}.

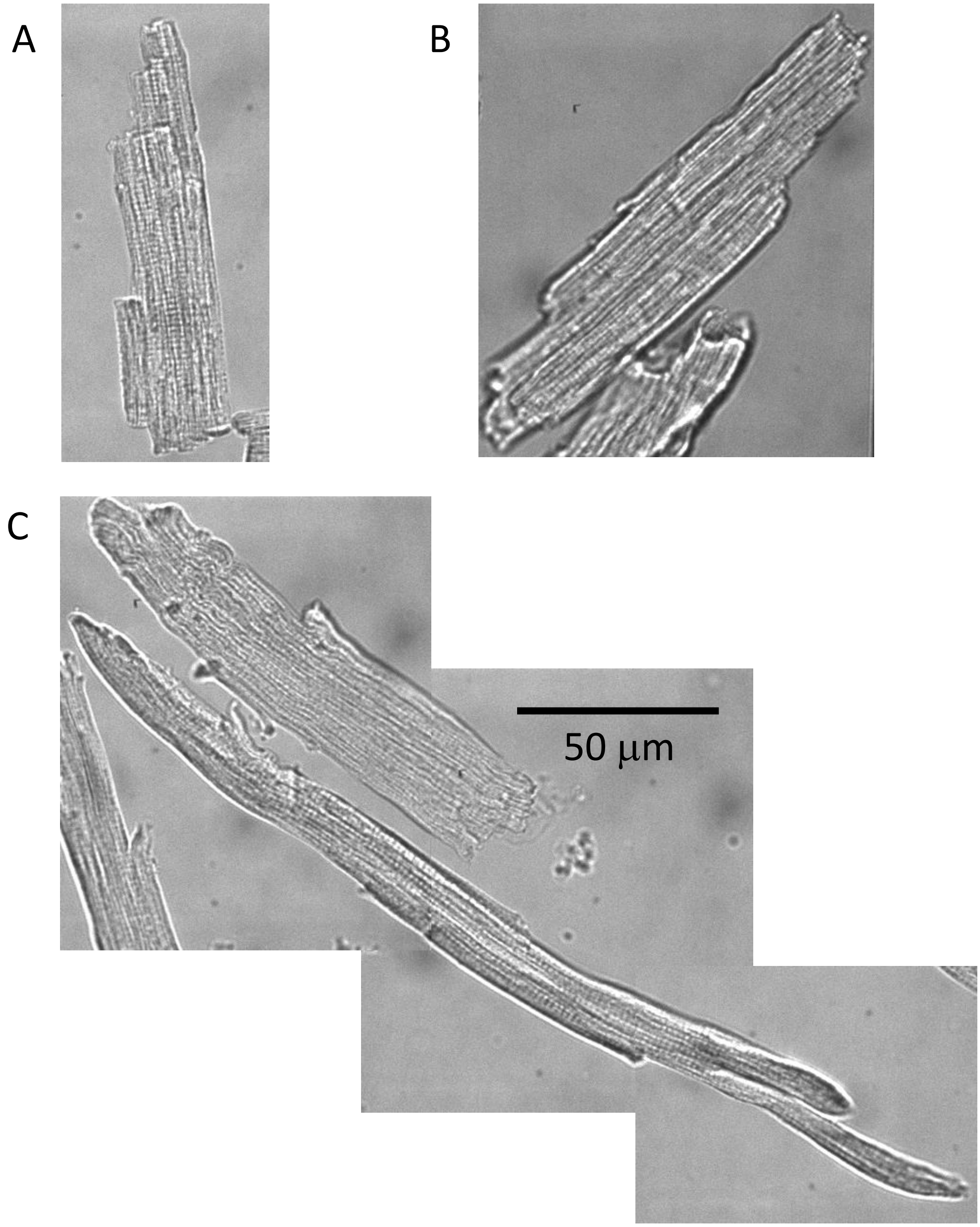

Figure S4. Typical images of myocytes obtained with 60x objective lens. ***A.*** WT, ***B.*** Candesartan treated DCM. ***C.*** Control DCM.
